# How to Get Sick

**Published:** 1876-05

**Authors:** 


### HOW TO GET SICK.

R. H. M. writes: "I slept in a room one night which had been washed out during the day, and was not quite dry. I awoke in the morning with a sore throat, which has continued ever since." The result is, he has had to abandon his theological studies, and place himself under treatment, after having lost two years' time.

H. M. writes: "I went to sleep, in a warm day, on the top of an ice-box, and have never been well since." He shortly after died of consumption.

T. H. took a very severe cold, conversation was laborious, but he had an appointment to preach, and "felt as if" he must fulfill it. He made the attempt, but utterance was attended with a pricking pain in the throat, and then a dull hurting came on in the throat, with subsequent "hemming," and fruitless "clearing." He was permanently disabled.

A modest man walked until quite fatigued, and perspiring freely, entered an omnibus, and sat next a lady, who opened the window to get some fresh air for herself. He soon became chilled, and was ill for three weeks.

H. P. got up at night and hoisted the window to look at a burning house, the cold air darted in on the unprotected body, just from a warm bed. A twelvemonth's illness resulted in dropsy.

A strong, hearty man came home on a hot summer's day, immediately took off his coat and hat, and sat in the open window, looking out upon a beautiful garden, over which the ocean breezes came to fan him. Before he was aware of it, he was chilled, was attacked with inflammation of the lungs, and died within a week.

A delicate young lady, an invalid, a patient of ours, in an excursion with several others, was "overtaken" by nightfall, and by a young gentleman. They were in a boat, and the boat was in the mud, the tide having gone out on a visit to the sea, and "there they were," a mile from shore, and several miles from home, that mile was extraordinarily long and short. Only think of it! A whole mile over a Jersey flat! covered with water, mud, and bulrushes, carrying in the arms, a young eighteen, with one of the sweetest voices, and faces, and forms, to be found in or out of Jersey. Bespattered with mud, dripping with fog, and dew, and slush, steaming with perspiration, and wearied with hunger, thirst, and fatigue, delighted and excited by the "*novelty of the thing*," they reached home at midnight The next day she "did—n't have anything the matter with her at all!" Why? She had taken lessons of us. Instead of pulling off her bonnet and shawl, and sitting in a cool place, or instead of undressing at once, and thus letting the air and cool linen sheets check the perspiration, she went into a warm room, closed all the doors and windows, sat some moments, laid aside the garments one by one, at intervals, and when cooled off, in the course of half an hour, retired to sound sleep and a healthful awaking.

A gentleman was in the habit of walking a mile to his office. The first thing was to draw off his boots and put on his slippers, without warming them. Every time he did it, there was an unpleasant shock of coldness; in a week he had rheumatic fever; every step he took was in pain. When he woke up of a morning, the whole body felt as if it had been pounded; he could not turn his body in any direction without acute suffering. He was sick three weeks, but afterwards always warmed his feet andslippers too, before he put them on; and in ten years he had no more of the "*Dengue*," or break-bone fever.

Another man rode three miles with a little child sleeping in his lap, which, pressing against the stomach, caused unusual warmth there. It was a chill, raw, November evening. In walking a hundred yards to the house, the child moving slowly and wind blowing, the whole abdomen was chilled in a moment. The next morning he awoke with the ominous pains of peritoneal inflammation, which is often fatal in three or four days.

Another person has invariably the same symptoms, if he sleeps on a spring-seat sofa, covered with hair-cloth, as the heat is quickly extracted from the body by that rapid conductor.

A hale, hearty man came home after a long day's ride and fasting; his tidy and affectionate wife prepared a delightful supper, prompt, warm, and abundant. He did it full justice. He said he had never enjoyed a meal so much before. The next morning he was dead.

A man had some accounts to draw off in mid-winter. It was a cold, damp night. He was greatly interested; time went, and the fire too. He felt a little chilly, but thought he would soon be done, and that it was not worth while to rebuild the fire. It was near one o'clock before he left for home, and he reached it most thoroughly chilled. Next morning, he had *Pneumonia*, and never got well.

Remaining at rest for hours in a cold room, in raw, cold, damp weather, is enough to kill three men out of four, by bringing on congestion of the lungs, lung fever, or inflammation of the lungs. Clergymen and lawyers often sacrifice their lives by speaking in warm rooms for some hours; the body, debilitated by the effort; the skin in a state of perspiration; the lungs all heated up; and thus, hungry, tired, and depressed in body and mind, go out into the cold air to ride or walk home—and to die in the very bloom of health and manhood. And yet, to know these little things, there are multitudes who hesitate to give a dollar a year, when on the knowledge of them, human life is daily hung, and for want of it, is daily lost.

